# Alternative splicing of a single exon causes a major impact on the affinity of *Caenorhabditis elegans* tropomyosin isoforms for actin filaments

**DOI:** 10.3389/fcell.2023.1208913

**Published:** 2023-09-07

**Authors:** Shoichiro Ono, Eichi Watabe, Keita Morisaki, Kanako Ono, Hidehito Kuroyanagi

**Affiliations:** ^1^ Departments of Pathology and Cell Biology, Emory University School of Medicine, Atlanta, GA, United States; ^2^ Winship Cancer Institute, Emory University School of Medicine, Atlanta, GA, United States; ^3^ Laboratory of Gene Expression, Graduate School of Biomedical Sciences, Tokyo Medical and Dental University, Tokyo, Japan; ^4^ Department of Biochemistry, University of the Ryukyus Graduate School of Medicine, Okinawa, Japan

**Keywords:** actin-binding proteins, coiled-coil, cytoskeleton, molecular dynamics simulation, nematodes

## Abstract

Tropomyosin is generally known as an actin-binding protein that regulates actomyosin interaction and actin filament stability. In metazoans, multiple tropomyosin isoforms are expressed, and some of them are involved in generating subpopulations of actin cytoskeleton in an isoform-specific manner. However, functions of many tropomyosin isoforms remain unknown. Here, we report identification of a novel alternative exon in the *Caenorhabditis elegans* tropomyosin gene and characterization of the effects of alternative splicing on the properties of tropomyosin isoforms. Previous studies have reported six tropomyosin isoforms encoded by the *C. elegans lev-11* tropomyosin gene. We identified a seventh isoform, LEV-11U, that contained a novel alternative exon, exon 7c (E7c). LEV-11U is a low-molecular-weight tropomyosin isoform that differs from LEV-11T only at the exon 7-encoded region. *In silico* analyses indicated that the E7c-encoded peptide sequence was unfavorable for coiled-coil formation and distinct from other tropomyosin isoforms in the pattern of electrostatic surface potentials. *In vitro*, LEV-11U bound poorly to actin filaments, whereas LEV-11T bound to actin filaments in a saturable manner. When these isoforms were transgenically expressed in the *C. elegans* striated muscle, LEV-11U was present in the diffuse cytoplasm with tendency to form aggregates, whereas LEV-11T co-localized with sarcomeric actin filaments. Worms with a mutation in E7c showed reduced motility and brood size, suggesting that this exon is important for the optimal health. These results indicate that alternative splicing of a single exon can produce biochemically diverged tropomyosin isoforms and suggest that a tropomyosin isoform with poor actin affinity has a novel biological function.

## Introduction

Actin plays essential roles in a number of cell biological processes by adapting to different subpopulations of actin cytoskeleton with various architecture and dynamics. Tropomyosin (Tpm) is one of important actin regulators that generate specific cytoskeletal environments in cells (Gunning et al., 2015; Manstein and Mulvihill, 2016; Hitchcock-DeGregori and Barua, 2017; Hardeman et al., 2020). Tropomyosin is generally known as an actin-filament-binding protein that regulates actomyosin interaction and actin filament stability. In mammals, at least 29 Tpm isoforms are expressed from four genes with extensive alternative splicing (Barua et al., 2011; Schevzov et al., 2011; Geeves et al., 2015). Most of cell types express multiple Tpm isoforms, and some of them are localized to distinct subcellular compartments and generate subpopulations of actin filaments with specific roles (Bryce et al., 2003; Grenklo et al., 2008; Tojkander et al., 2011; Gateva et al., 2017). Additionally, *Drosophila* has several Tpm isoforms that localize to subcellular regions where actin is not typically associated (Erdelyi et al., 1995; Goins and Mullins, 2015; Cho et al., 2016; Veeranan-Karmegam et al., 2016). In particular, *Drosophila* Tm1-I/C is an atypical isoform that contains large intrinsically disordered domains and forms intermediate filament-like polymers (Cho et al., 2016; Veeranan-Karmegam et al., 2016; Sysoev et al., 2020) with an antiparallel coiled coil (Dimitrova-Paternoga et al., 2021) to regulate mRNA transport (Veeranan-Karmegam et al., 2016; Gaspar et al., 2017; Dimitrova-Paternoga et al., 2021). Therefore, further functional characterization of Tpm isoforms is needed to understand the mechanism of isoform-specific actin regulation and explore an actin-independent function of certain isoforms.

The nematode *Caenorhabditis elegans* has a single Tpm gene, *lev-11*, that was originally identified from a screen for mutants with resistance to levamisole, an agonist of the acetylcholine receptor (Lewis et al., 1980). Severe *lev-11* mutations cause paralysis and developmental arrest at a late embryonic stage (Williams and Waterston, 1994). The *lev-11* gene regulates muscle contractility and myofibril assembly (Ono and Ono, 2002; Yu and Ono, 2006; Hwang et al., 2016; Barnes et al., 2018; Ono et al., 2022), development of neuromuscular junctions (muscle arms) (Dixon and Roy, 2005), ovulation by the somatic gonad (Ono and Ono, 2004), and male mating behavior (Gruninger et al., 2006). At least six Tpm isoforms are expressed from the *lev-11* gene by two separate promoters and alternative splicing (Kagawa et al., 1995; Anyanful et al., 2001; Barnes et al., 2018; Watabe et al., 2018). Alternative splicing of seventh exons, exon 7a (E7a) and exon 7b (E7b), is differentially regulated in the head and body regions of the body wall muscles and required for proper regulation of muscle contractility in the specific subset of muscle cells (Barnes et al., 2018). However, E7a- and E7b-encoded sequences do not alter actin-binding properties of the Tpm isoforms (Barnes et al., 2018), and how alternative splicing of the *lev-11* gene affects the biochemical properties of the Tpm isoforms remains unknown. In this study, we report identification of a third alternative exon 7 that causes a strong negative impact on the actin-binding properties of a Tpm isoform. Identification of a Tpm isoform with poor actin affinity suggests that it is involved in a biological process that has not been recognized for conventional Tpm isoforms.

## Results

### Identification of a new tropomyosin isoform containing a novel alternative exon 7 of the lev-11 gene

**FIGURE 1:**
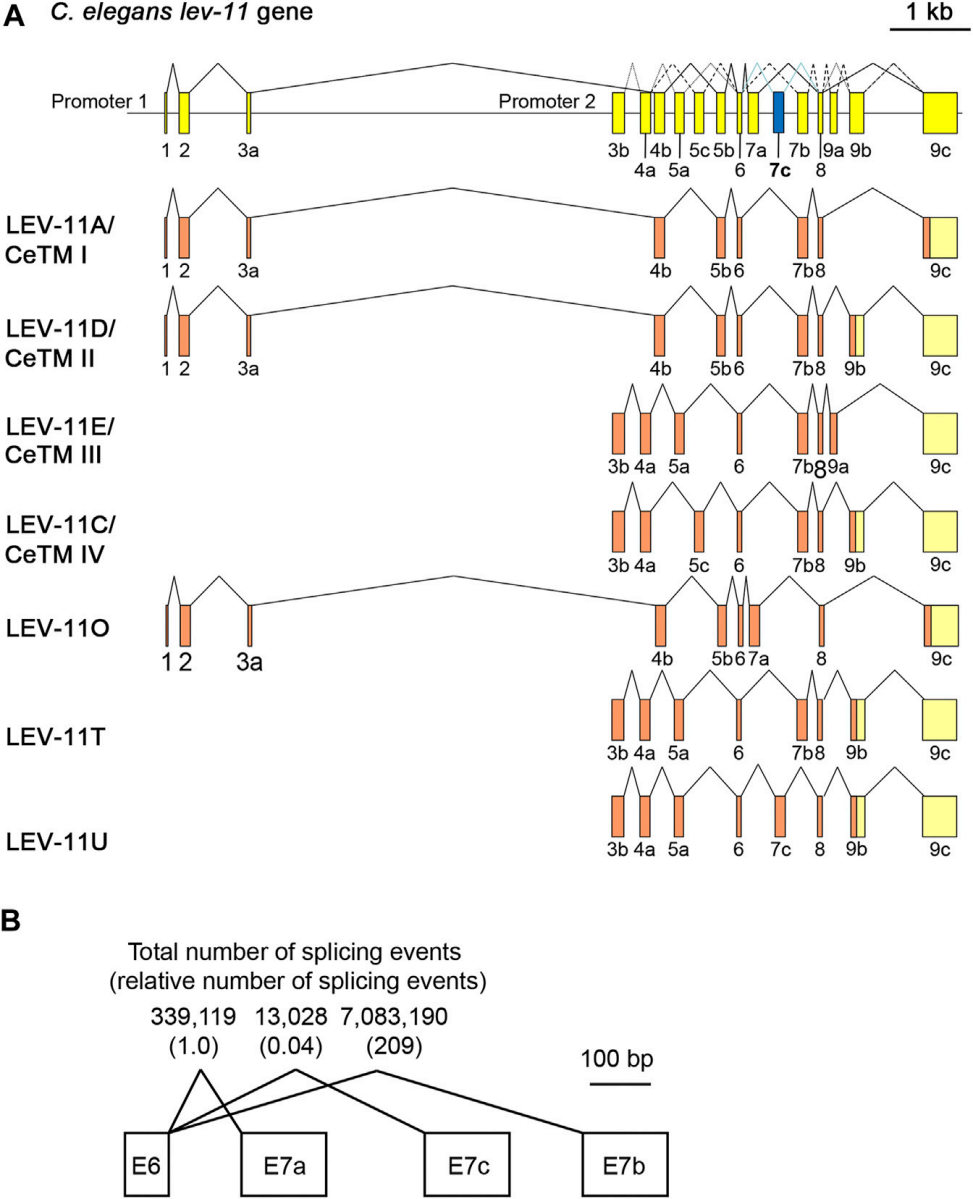
LEV-11U is a seventh *C. elegans* tropomyosin isoform containing a third alternative exon 7. **(A)**, Schematic representation of the *lev-11* gene structure (top) with numbered boxes indicating exons, and splicing patterns of LEV-11 isoforms (bottom). The newly identified exon 7c (E7c) is shown in blue. Coding and noncoding regions are shown in orange and light yellow, respectively. **(B)** Total number of splicing events from the RNA-seq data that had been reported in WormBase (release WS289; www.wormbase.org). Numbers in parentheses are relative numbers of splicing events.

Using reverse transcription-polymerase chain reaction and cDNA cloning from *C. elegans* RNA, we identified a cDNA clone encoding a new low-molecular-weight Tpm isoform containing an exon 7 sequence that has not been recognized as an exon in previous studies or any sequence databases including WormBase (Davis et al., 2022). This is the third alternative exon 7 and designated as exon 7c (E7c) (Figure 1). The new Tpm isoform, which we designated as LEV-11U (GenBank accession number: OQ473578), contained an exon combination of E3b-E4a-E5a-E6-E7c-E8-E9b (Figure 1A). Therefore, LEV-11U is identical to LEV-11T (E3b-E4a-E5a-E6-E7b-E8-E9b) (GenBank accession number: LC215398) (Watabe et al., 2018) except for the exon 7 sequence (Figure 1A). The accumulated RNA-seq data in WormBase (release WS289) indicate that E7b is the most frequently selected exon, but E7a (∼1/200 of E7b) and E7c (∼1/5000 of E7b) are rarely selected exons (Figure 1B). Tissue and cell distribution of the E7c selection is currently under investigation.

**FIGURE 2:**
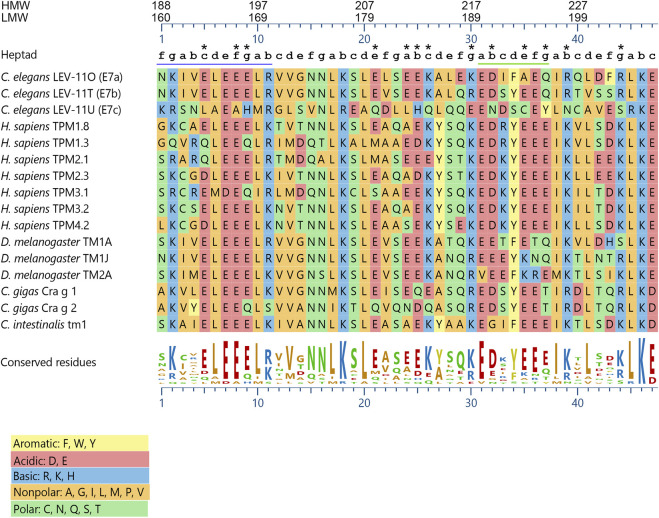
The *lev-11* E7c encodes a unique peptide sequence. The peptide sequences encoded by the alternative exon 7s of the *C. elegans lev-11* gene were aligned with the equivalent sequences of Tpm isoforms from various species: *Homo sapiens*- TPM1.8 (NCBI Reference Sequence: NP_001288218.1), TPM1.3 (NCBI Reference Sequence: NP_001018020.1), TPM2.1 (NCBI Reference Sequence: NP_998839.1), TPM2.3 (NCBI Reference Sequence: NP_001288155.1), TPM3.1 (NCBI Reference Sequence: NP_705935.1), TPM3.2 (NCBI Reference Sequence: NP_001036816.1), and TPM4.2 (NCBI Reference Sequence: NP_003281.1); *Drosophila melanogaster*- TM1A (NCBI Reference Sequence: NP_524360.2), TM1J (NCBI Reference Sequence: NP_732004.1), and TM2A (NCBI Reference Sequence: NP_524361.4); *Crassostrea gigas*- Cra g 1 (NCBI Reference Sequence: NP_001354222.1) and Cra g 2 (NCBI Reference Sequence: NP_001295835.2); and *Ciona intestinalis*- Ctm1 (GenBank accession number: CAA45469.1). Alignment and sequence conservation were analyzed using Clustal W (Thompson et al., 1994) with MegAlign Pro 17 (DNASTAR, Inc.). Residue numbers for high-molecular-weight (HMW) and low-molecular-weight (LMW) isoforms are indicated at the top. Positions within heptads (*a-g*) are indicated above the sequences. The underlined regions indicate (*left*) the region where strong positive surface charges are found in *C. elegans* LEV-11U (E7c) (see Figure 4), and (*right*) the heptads with highly variable probabilities of coiled-coil formation (see Figure 3A). Asterisks indicate positions where charged (acidic and basic) residues are not conserved in *C. elegans* LEV-11U (E7c). Charged residues are considered as “conserved” when more than eight Tpm isoforms contain residues of the same charges.

Alignment of the exon 7-encoded sequences showed that the E7c-encoded sequence was distinct from E7a- and E7b-encoded sequences (Figure 2; Table 1). Although E7a and E7b encode 78.7% identical amino acid sequences, the E7c-encoded sequence is only 17.0% and 19.2% identical to E7a- and E7b-encoded sequences, respectively (Figure 2; Table 1). By comparing with the equivalent sequence of human Tpm1.8 (also known as Tpm5 or Tpm1.8cy) (residues 188–234; NCBI Reference Sequence: NP_001288218.1), E7a- and E7b-encoded sequences are 55.3% and 59.6% identical, respectively, but E7c-encoded sequence is only 17.0% identical (Figure 2; Table 1), suggesting that the E7c-encoded polypeptide is biochemically different from other equivalent polypeptides.

**TABLE 1:** Amino acid sequence identity (%) in the exon 7-encoded regions of representative Tpm isoforms.

	*C. elegans* LEV-11O (E7a)	*C. elegans* LEV-11T (E7b)	*C. elegans* LEV-11U (E7c)	*H. sapiens* TPM1.8	*D. melanogaster* TM1A	*C. gigas* cra g 1	*C. intestinalis* tm1
*C. elegans* LEV-11O (E7a)	-	78.7	17.0	55.3	74.5	59.6	57.5
*C. elegans* LEV-11T (E7b)		-	19.2	60.0	72.3	68.1	55.3
*C. elegans* LEV-11U (E7c)			-	17.0	19.2	12.8	12.8
*H. sapiens* TPM1.8				-	59.6	55.3	72.3
*D. melanogaster* TM1A					-	55.3	59.6
*C. gigas* Cra g 1						-	51.1
*C. intestinalis* tm1							-

Further comparison of the E7c-encoded sequence with the equivalent sequences of Tpm isoforms from various species (Figure 2) also suggested its unique properties. The three exon 7-encoded sequences from the *C. elegans lev-11* gene were aligned with equivalent sequences of Tpm isoforms from human (*Homo sapiens*), fruit fly (*Drosophila melanogaster*), pacific oyster (*Crassostrea gigas*), and ascidian (*Ciona intestinalis*). The sequence comparison showed that eight basic (R, K, and H) and fourteen acidic (D and E) residues were evolutionarily conserved among the examined species (Figure 2), because many of these conserved charged residues are important for actin binding or actomyosin regulation (Barua et al., 2011; Barua et al., 2012; Barua et al., 2013). Although most of these charged residues were conserved in the E7a- and E7b-encoded sequences, four basic and nine acidic residues were not conserved in the E7c-encoded sequence (Figure 2, asterisks). Since charged amino acids are often involved in regulating the structure and function of proteins, the E7c-encoded polypeptide might have distinct biochemical properties from other Tpm isoforms.

### Exon 7c encodes a peptide sequence that is unfavorable for coiled-coil formation

**FIGURE 3:**
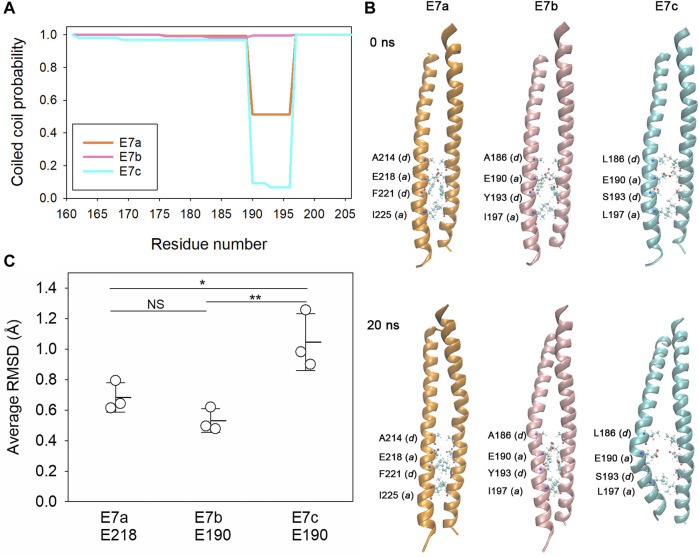
The E7c-encoded peptide is predicted to be unfavorable for coiled-coil formation. **(A)**, Probability of coiled-coil formation was calculated from the full-length sequences of LEV-11O (E7a: orange), LEV-11T (E7b: pink) and LEV-11U (E7c: light blue) by COILS, and plots of the exon 7-encoded regions are shown. For simplicity, only residue numbers for low-molecular-weight isoforms are shown. Residues 190–196 are highly variable in coiled-coil probability and underlined in Figure 2. **(B)**, Molecular dynamics simulations of the exon 7-encoded regions. Energy-minimized model structures of the E7a-, E7b-, and E7c-encoded regions (0 ns, top) were subjected to 20 ns of molecular dynamics simulations (bottom). Core residues (positions *a* and *d*) near residue 190 (218 in E7a because no low-molecular-weight isoform containing E7a has been identified) are labeled, where the most noticeable structural changes were observed. **(C)**, Structural deviations of E218 of E7a and E190 of E7b and E7c were quantified from three independent simulations of 40 ns as average RMSD (Å) as described in Experimental Procedures. *, 0.01 < *p* < 0.05; ***, *p* < 0.001.

The biophysical properties of the E7-encoded sequences were analyzed using *in silico* approaches. Tropomyosins are generally 100% α-helical polypeptides forming coiled-coil dimers (Hitchcock-DeGregori and Barua, 2017). Prediction of coiled-coil formation by COILS (Lupas et al., 1991) indicated nearly 100% probability for the E7b-encoded sequence but medium and low probabilities for the E7a- and E7c-encoded sequences, respectively, in a heptad at residues 190–196 (Figure 2; Figure 3A). To examine the structural stability of the E7-encoded polypeptides, a coiled-coil dimer of each E7-encoded sequence was analyzed by molecular dynamics (MD) simulations (Figures 3B,C). The E7a- and E7b-encoded polypeptides maintained relatively stable coiled-coils (Figure 3B), whereas the E7c-encoded polypeptide was readily unwound at around E190 (in the LEV-11U sequence) resulting in a separation of the two strands (Figure 3B). Quantitative analysis of root-mean-square deviation (RMSD) values indicates that residue 190 of LEV-11U (E7c) was more significantly displaced from the initial position than the equivalent residues of LEV-11O (E7a) and LEV-11T (E7b) (Figure 3C). It should be noted that our simulations of 40 ns may be too short, and that longer simulations of the E7-encoded sequences and full-length proteins may be needed to predict how the E7-encoded sequences contribute to the biophysical properties of the Tpm isoforms. These results suggest that LEV-11U has a region that is unfavorable to maintain a coiled-coil within the E7c-encoded sequence.

### Distribution of electrostatic potentials is unique in the E7c-encoded polypeptide

**FIGURE 4:**
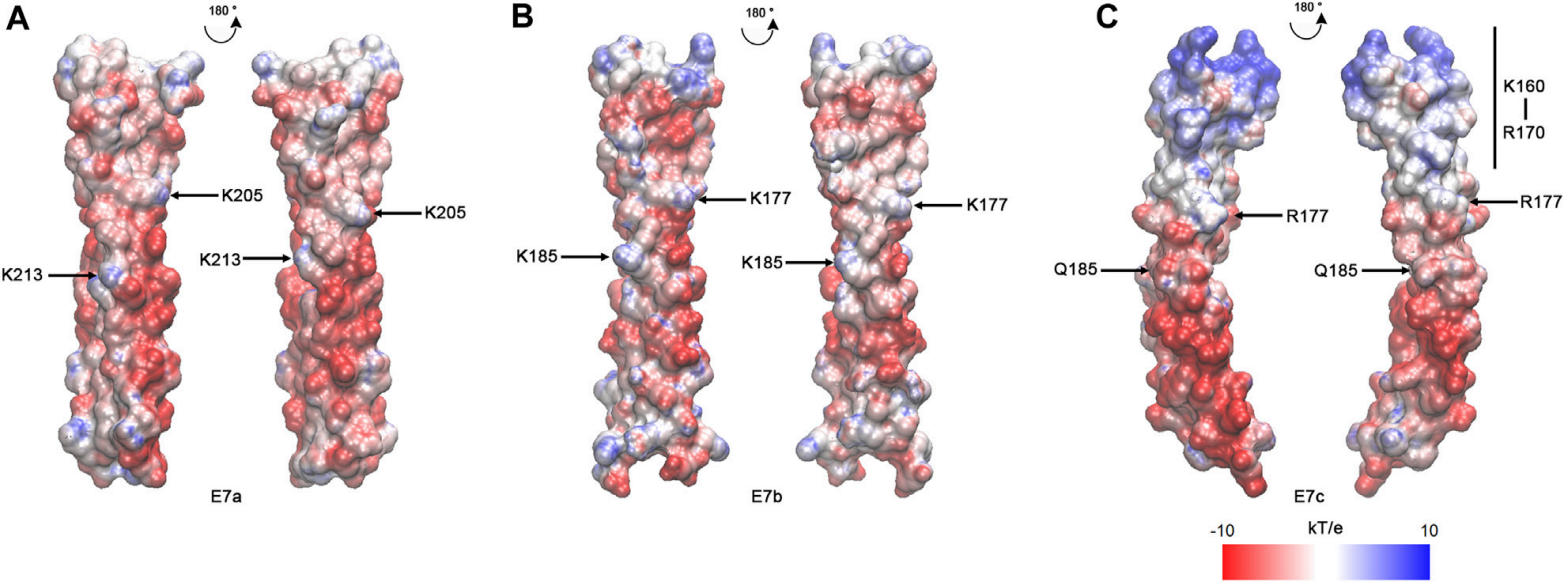
The E7c-encoded peptide has a distinct pattern of electrostatic surface potentials from the E7a- and E7b-encoded peptide. The electrostatic surface potentials of E7a- **(A)**, E7b- **(B)**, and E7c- **(C)** encoded polypeptides after MD simulations are shown with red-white-blue coloring, ranging from −10 to 10 kT/e. K205 and K213 in rat skeletal muscle Tpm have been shown to be involved in F-actin binding (Barua et al., 2013), and equivalent residues (K205 and K213 in E7a; K177 and K185 in E7b; R177 and Q185 in E7c) are marked. The N-terminal region of E7c (K160 - R170) has strong positive charges (underlined in Figure 2).

As suggested by the sequence alignment (Figure 2), the E7c-encoded polypeptide was predicted to be distinct from the E7a- and E7b-encoded polypeptides in the pattern of electrostatic surface potentials (Figure 4). After the MD simulations, electrostatic surface potentials of each polypeptide were estimated using Adaptive Poisson-Boltzmann Solver (APBS) (Jurrus et al., 2018). Although the E7a- and E7b-encoded polypeptides were dominated by negative charges with scattered small patches of positive charges on their surfaces (Figures 4A,B), the E7c-encoded polypeptide had a large patch of positive charges at the N-terminal portion at K160–R170 (Figure 4C, underlined in Figure 2). This region corresponds to a second half of the fifth actin-binding period (Barua et al., 2011; Barua, 2013), but how this region contributes to actin binding is not understood. Nonetheless, the difference in the surface charges suggest that the E7c-encoded polypeptide may have unique properties in interacting with other proteins.

### LEV-11U, containing exon 7c, binds poorly to actin filaments

**FIGURE 5:**
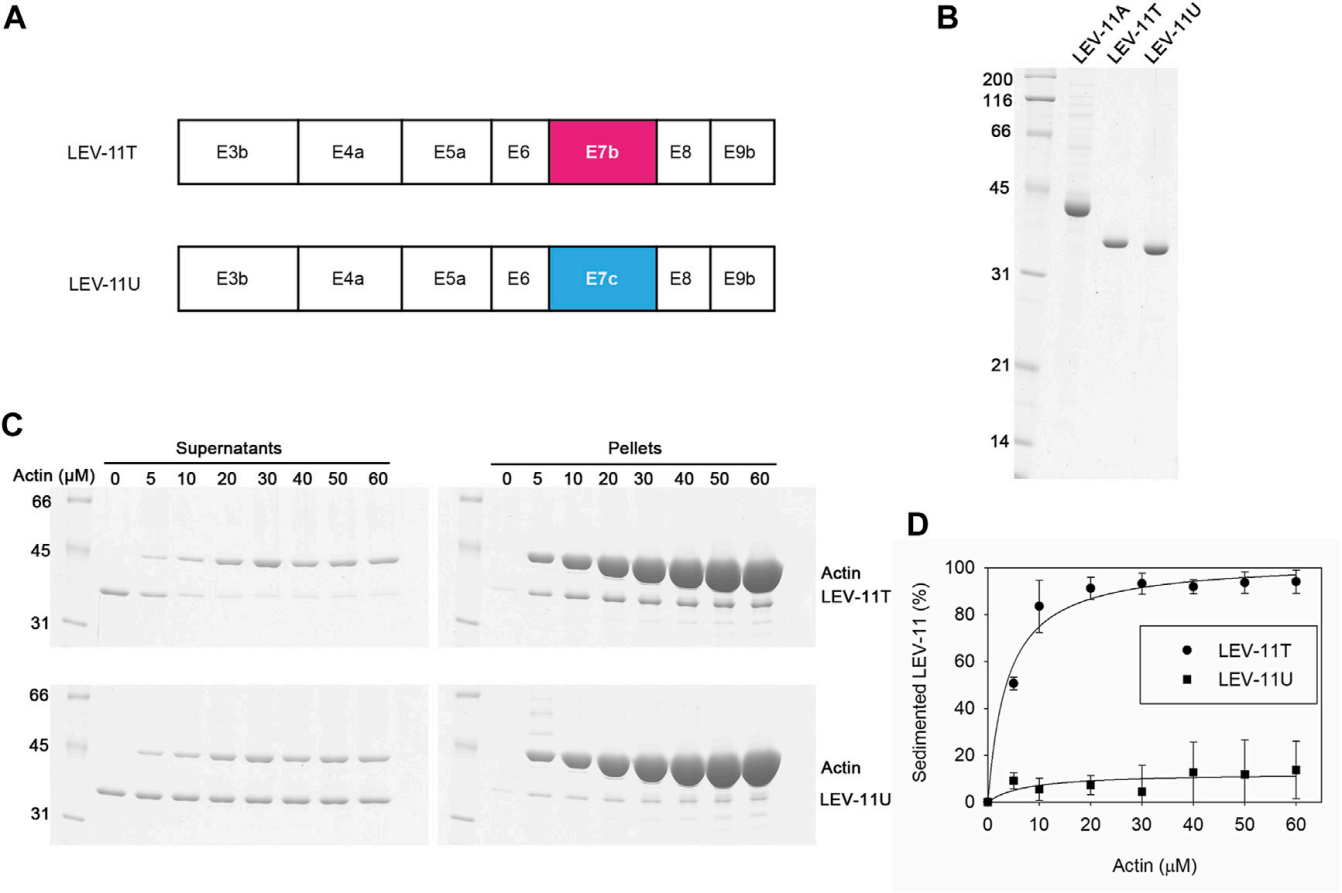
LEV-11U binds poorly to actin filaments *in vitro*. **(A)**, Exon combinations of LEV-11T and LEV-11U highlight their difference only in the exon 7-encoded regions. **(B)**, Bacterially expressed and purified LEV-11A, LEV-11T, and LEV-11U (0.5 μg each) were analyzed by SDS-PAGE and Coomassie staining. LEV-11A was included as a representative high-molecular-weight isoform for comparison with the low-molecular-weight isoforms, LEV-11T and LEV-11U. Molecular mass markers in kDa are shown on the left. **(C)**, F-actin co-sedimentation (supernatant depletion) assays. LEV-11T or LEV-11U (2 μM) was incubated with 0–60 μM F-actin for 1 h and ultracentrifuged. Supernatants and pellets were separated and examined by SDS-PAGE. Positions of LEV-11T, LEV-11U, and actin are indicated on the *right*. **(D)**, Quantitative analysis of the F-actin co-sedimentation assays. Percentages of sedimented LEV-11T (circles) or LEV-11U (squares) were quantified and plotted as a function of actin concentrations. Three independent experiments were performed and plotted as average ±standard deviation.

Previously, we have reported that the choice of E7a or E7b in high-molecular-weight Tpm isoforms does not significantly alter their actin-binding properties (Barnes et al., 2018). To determine the effects of the E7c-encoded sequence on the biochemical properties of Tpm isoforms, we compared LEV-11T and LEV-11U, the two low-molecular-weight isoforms that differ only at the E7-encoded regions (Figure 5A). Both isoforms were bacterially expressed with extra Ala-Ser after the initiator Met to mimic N-terminal acetylation (Monteiro et al., 1994), purified to homogeneity (Figure 5B), and tested for actin-filament binding using F-actin co-sedimentation assays. Typically, we perform the F-actin co-sedimentation assays by using a constant concentration of F-actin and varying concentrations of Tpm, and quantify Tpm in the pellets (Ono and Ono, 2002; Barnes et al., 2018). Instead, we performed the assays using a constant concentration of a LEV-11 isoform and varying concentrations of F-actin and quantified depletion of a LEV-11 isoform from the supernatants to determine the bound portions, because LEV-11U bound poorly to F-actin and non-specifically precipitated portions of LEV-11U obscured its specific co-sedimentation with F-actin. Constant concentrations (2 μM) of LEV-11T or LEV-11U were incubated with increasing concentrations (0–60 μM) of F-actin and fractionated into supernatants and pellets after ultracentrifugation. Under these conditions, LEV-11T, containing the E7b-encoded sequence, was increasingly shifted from the supernatant to pellet fractions as F-actin concentration was increased (Figure 5C, top), indicating that LEV-11T specifically bound to F-actin. Binding of LEV-11T to F-actin was saturable with an estimated dissociation constant (*K*_*d*_) of 3.7 ± 0.64 μM (n = 3) (Figure 5D). In contrast, the majority of LEV-11U, containing the E7c-encoded sequence, remained in the supernatants regardless of the F-actin concentrations (Figure 5C, bottom), indicating that LEV-11U bound poorly to F-actin with no measurable affinity (Figure 5D). Since LEV-11T and LEV-11U differ only in the E7-encoded sequences, these results demonstrate that the choice of alternative E7s confers a major difference in the actin affinity of the Tpm isoforms.

**FIGURE 6:**
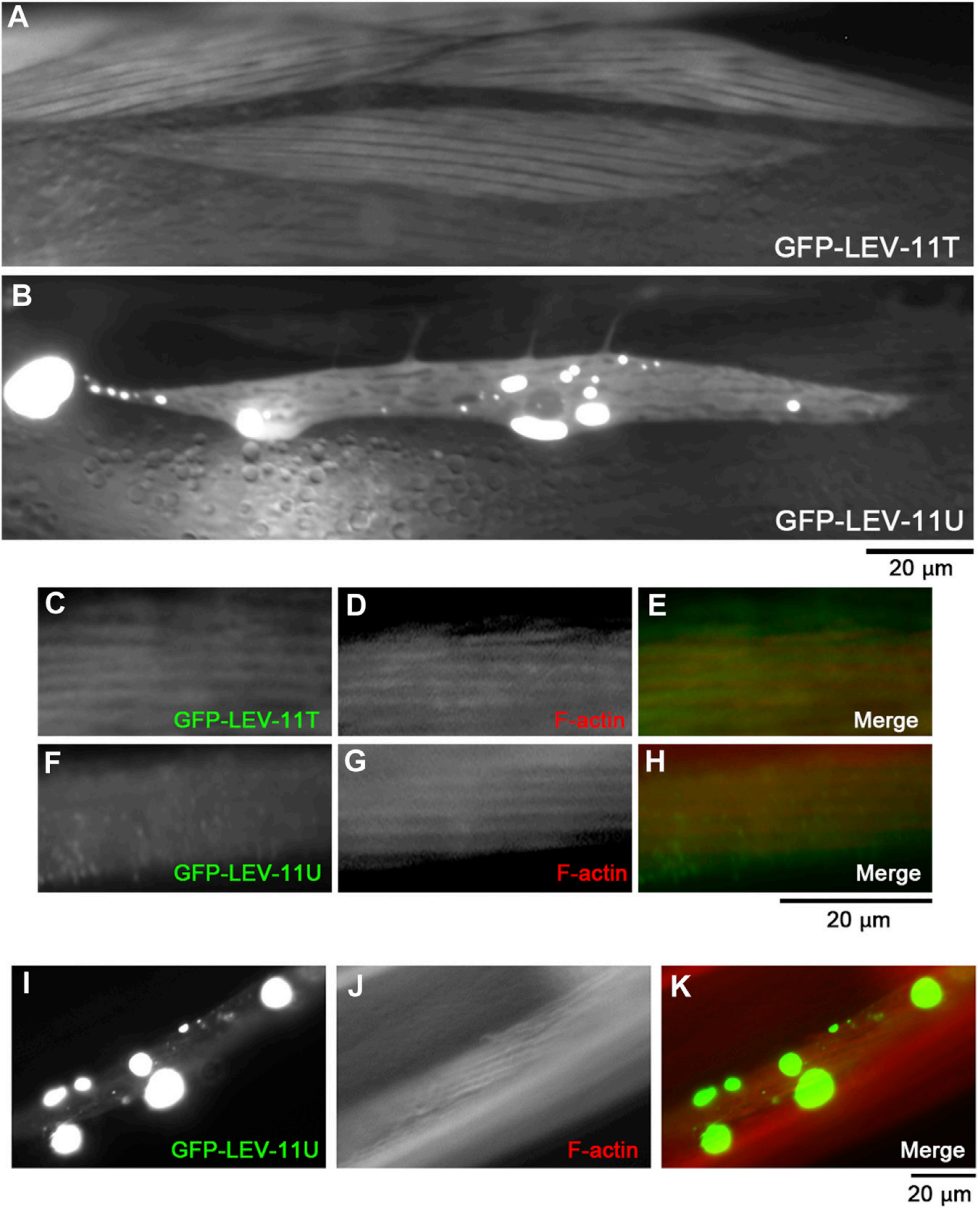
LEV-11T, but not LEV-11U, can interact with sarcomeric actin filaments in the *C. elegans* body wall muscle. **(A, B)**, Fluorescent images of live worms expressing GFP-LEV-11T **(A)** or GFP-LEV-11U **(B)** in the body wall muscle. **(C–H)**, Worms expressing GFP-LEV-11T **(C)** or GFP-LEV-11U **(F)** were fixed and stained for actin filaments with ATTO594-labeled phalloidin **(D, G)**. Merged images are shown in **(E**, **H)** (GFP in green and F-actin in red). **(I–K)**, A worm expressing a high level of GFP-LEV-11U **(I)** was stained for actin filaments with ATTO594-labeled phalloidin **(J)**. Merged image is shown in **(K)** (GFP in green and F-actin in red). Bars, 20 μm.

To determine whether LEV-11T and LEV-11U can bind to actin *in vivo*, these Tpm isoforms were tagged with green fluorescent protein (GFP) and expressed transgenically in the body wall muscle cells (Figure 6). Although low-molecular-weight Tpm isoforms are typically not enriched in muscle cells, highly ordered sarcomeric actin organization allows us to determine colocalization of the Tpm isoforms with F-actin *in vivo*. In live worms without fixation, GFP-LEV-11T localized in a striated pattern (Figure 6A), whereas GFP-LEV-11U was present in the diffuse cytoplasm and often concentrated in aggregates of various sizes at random locations within the cytoplasm (Figure 6B). These aggregates are likely the result of overexpression because such structures have not been detected by immunofluorescent staining of endogenous Tpm using a polyclonal antibody that was raised against the whole Tpm protein (Ono and Ono, 2002; Ono et al., 2022). When these worms were fixed and stained with fluorescently labeled phalloidin, GFP-LEV-11T colocalized with F-actin in a striated manner (Figures 6C–E), but GFP-LEV-11U was diffusely present in the cytoplasm and did not exhibit a striated pattern (Figures 6F–H). Furthermore, when GFP-LEV-11U was overexpressed and aggregated, F-actin was not accumulated in the aggregates of GFP-LEV-11U, and striated organization of F-actin was not significantly affected (Figures 6I–K), suggesting that association of GFP-LEV-11U with F-actin was negligible in the muscle cells. Therefore, these results indicate that *in vivo* localization of LEV-11T and LEV-11U can be significantly different, which could be due to the difference in their affinity for F-actin.

### Exon 7c mutation causes mild reduction in worm motility and brood size

**FIGURE 7:**
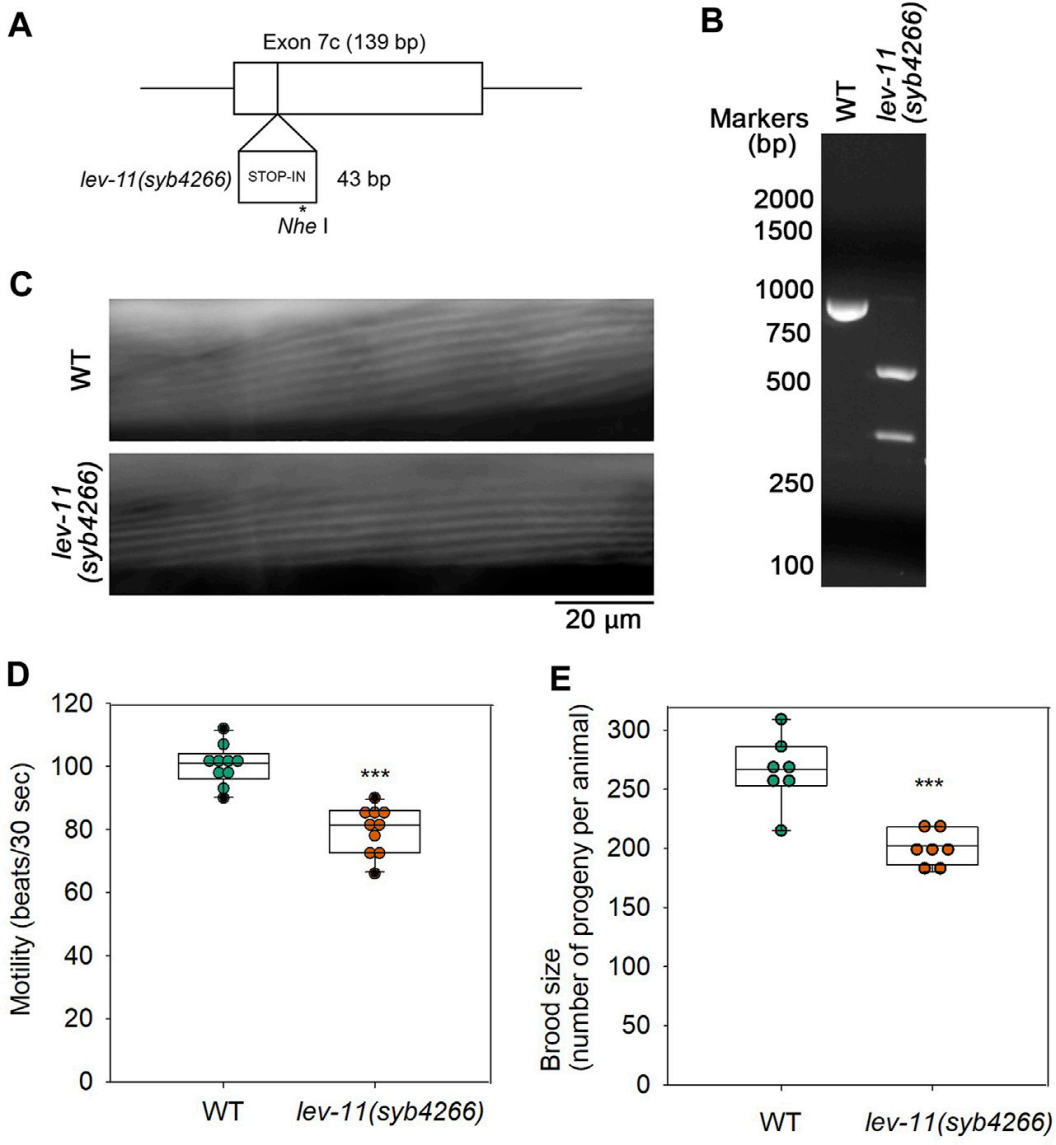
Mutation in E7c causes mild reduction in worm motility and brood size without affecting sarcomeric actin organization. **(A)**, A STOP-IN cassette of 43 bp was inserted in E7c to generate *lev-11(syb4266)*. An asterisk indicates a unique *Nhe* I site. **(B)**, Genotyping of wild-type (WT) and *lev-11(syb4266)* was done by performing PCR amplification of a genomic DNA fragment containing E7c and digestion by *Nhe* I followed by agarose electrophoresis. The DNA fragment from WT (851 bp; left) was not cut, whereas that from *lev-11(syb4266)* (894 bp) was cut by *Nhe* I into two smaller fragments (548 and 346 bp; right). DNA size markers (Nacalai United States, catalog no. NU02002) are shown on the left of the gel image. **(C)**, Actin organization in WT (top) and *lev-11(syb4266)* (bottom) were examined by staining with ATTO594-labeled phalloidin, showing no detectable differences. Bar, 20 μm. **(D)**, Worm motility was quantified as beating frequency in liquid (beats per 30 s) (n = 10). **(E)**, Brood size was determined as number of progeny per animal (n = 7). In **(D, E)**, boxes represent the range of the 25th and 75th percentiles, with the medians marked by solid horizontal lines, and whiskers indicate the 10th and 90th percentiles. ***, *p* < 0.001.

To determine *in vivo* significance of E7c, we generated a mutant, *lev-11(syb4266)*, in which a STOP-IN cassette containing premature stop codons was inserted in E7c (Figure 7A). The STOP-IN cassette contains stop codons in all three reading frames (Wang et al., 2018) and is likely to cause nonsense-mediated decay of the transcripts containing premature stop codons. Therefore, *lev-11(syb4266)* should not express a LEV-11 Tpm isoform containing the E7c-encoded sequence. The STOP-IN cassette contains a unique *Nhe* I site (Figure 7A). Therefore, a PCR-amplified genomic DNA fragment could be cut by *Nhe* I only when the STOP-IN cassette was present (Figure 7B). The *lev-11(syb4266)* homozygotes were viable and showed no gross phenotypes. Sarcomeric organization of actin filaments in the body wall muscle was indistinguishable between wild-type and *lev-11(syb4266)* (Figure 7C). However, *lev-11(syb4266)* showed slightly slower worm motility (Figure 7D) and reduced brood size (Figure 7E) as compared with wild-type. Although the cellular basis of these phenotypes is currently unknown, these data suggest that the E7c-encoded sequence is required for optimal health of the worms.

## Discussion

Each alternative exon 7 encodes 47 amino acids, which is only 18% of total 256 amino acids of the *C. elegans* low-molecular-weight Tpm isoforms. Tpm generally has 6 or 7 actin-binding domains with a periodic pattern of charged amino acids serving as actin-binding interfaces (Barua et al., 2011; Barua et al., 2013). Therefore, it is surprising that a sequence variation within a small portion of a Tpm causes a major difference in actin affinity. The poor actin-binding properties of LEV-11U may be caused by the structurally unstable region of the E7c-encoded sequence around E190 (Figure 3). The glutamic acid residue is not typical for position *a* of a coiled-coil-forming heptad sequence, because residues at position *a* normally contribute to forming a hydrophobic core (Woolfson, 2023). Nonetheless, glutamic acid is highly conserved at the equivalent positions in Tpms across species (E218 in vertebrate striated muscle isoforms; see Figure 2) (Barua et al., 2011). A structural study of rabbit skeletal muscle α-Tpm demonstrated that the hydrophobic core around E218 (equivalent to E190 of LEV-11U) is disrupted by a water-bearing hole (Minakata et al., 2008). Similar core disruptions are also found at several locations in a Tpm dimer and are speculated to be required for structural flexibility to adapt to the surface of an actin filament (Brown et al., 2005; Minakata et al., 2008; von der Ecken et al., 2015; Hitchcock-DeGregori and Barua, 2017; Lehman et al., 2018). However, the structural disruption in the E7c-encoded region may cause excessive bending or flexibility of the LEV-11U dimer that may not be favorable for binding to actin filaments.

In addition, sequence variations at actin-binding residues and/or surfaces may affect the affinity for actin filaments. Variation in the charged residues makes a unique region of the E7c-encoded polypeptide with strong positive charges on the surface (Figures 2, 4), whereas the equivalent regions of the E7a- and E7b-encoded polypeptides are negatively charged (Figure 4). Mutations of charged residues into alanines in the equivalent region (a second half of period 5) of rat α-Tpm causes only a modest reduction in the actin affinity (Barua et al., 2011). Human Tpm2.1 is somewhat similar to the *C. elegans* E7c-encoded sequence containing two basic residues and lacking an acidic residue in the equivalent region (Figure 2). Intriguingly, human Tpm2.1 has lower affinity for F-actin than other Tpm isoforms (Janco et al., 2016). Therefore, the positive charges at this region may be repelled from the F-actin surface to reduce the affinity for F-actin. Although this is a small portion of the LEV-11U molecule, even single point mutations of Tpm can significantly reduce the actin affinity of the entire Tpm molecule (Barua et al., 2013; Racca et al., 2020). Therefore, the poor actin affinity of LEV-11U may be caused by variations in both coiled-coil structure and actin-binding interfaces.

Our discovery raises a major question regarding the function of a Tpm isoform with poor actin affinity. *In vivo* selection pattern of E7c is currently under investigation. Our preliminary experiments using a fluorescence reporter for E7c using a published method (Kuroyanagi et al., 2010; Barnes et al., 2018; Watabe et al., 2018) suggested that E7c was selected widely in muscle and non-muscle tissues (our unpublished data). However, the transgenic worms expressing the E7c reporter were lethargic, and we have not been able to obtain high-resolution images to determine the selection pattern. The poor actin affinity of LEV-11U suggests that this isoform may have an actin-independent function or hetero-dimerize with other Tpm isoform to “neutralize” the actin-binding properties. Alternatively, LEV-11U may still bind to actin filaments in a condition-dependent manner, such as post-translational modification of LEV-11U or actin, cooperation with other actin-binding proteins, and/or actin isoforms. A recent study demonstrated that recombinant tropomyosins expressed in mammalian cells contain a number of eukaryotic post-translational modifications and behave differently from bacterially expressed tropomyosins (Carman et al., 2021), suggesting that such modifications, in addition to primary sequences, can affect the actin-binding properties of tropomyosins. Although GFP-LEV-11U was present in the diffuse cytoplasm in *C. elegans* muscle cells, this result does not exclude possibility that LEV-11U binds to non-sarcomeric actin filaments in muscle. Future characterization of expression pattern and biochemical properties of LEV-11U should be important to understand the physiological function of this new Tpm isoform and to explain molecular and cellular bases of the phenotypes in the E7c mutant worms.

The finding of a novel alternative exon was also surprising. *Caenorhabditis elegans* was the first animal with the entire genome sequenced in 1998 (The *C. elegans* Sequencing Consortium, 1998), and its genome has been extensively studied for more than two decades. This raises possibility that unrecognized exons may still exist in other genes in *C. elegans* and also in other organisms. With the technical advancements in transcriptome analysis, additional new transcripts may be discovered, and some of them may have biologically important functions. Therefore, the *lev-11* tropomyosin gene in *C. elegans* can be a useful model to study the complexity of alternative splicing and functional diversity of the splice variants.

## Experimental procedures

### Worm culture

The worms were cultured following standard methods (Stiernagle, 2006). The N2 wild-type *C. elegans* strain was obtained from the *Caenorhabditis* Genetics Center (Minneapolis, MN).

### Characterization of LEV-11U cDNA

Clones for LEV-11U were discovered in the process of reverse-transcription PCR, cloning, and sequencing of low-molecular-weight Tpm isoforms as described previously (Watabe et al., 2018). The LEV-11U cDNA was subcloned into a pET-3d vector with additional codons for Ala-Ser (GCTAGC) after the initiator Met and used for expression of recombinant LEV-11U in *Escherichia coli*. Sequences were verified by DNA sequencing.

### Molecular dynamics simulations

CCBuilder Mk.2 (Wood and Woolfson, 2018) was used to generate structural models of parallel coiled-coil dimers of full-length LEV-11O, LEV-11T, and LEV-11U, which were then truncated into the E7-encoded regions: residues 188–234 for LEV-11O (E7a) and residues 160–206 for LEV-11T (E7b) and LEV-11U (E7c). Using Visual Molecular Dynamics (VMD) (Humphrey et al., 1996), each of these structures was solvated by a rectangular box of water particles and ionized at 0.1 M KCl. The layer of the box was set to 15 Å. Nanoscale Molecular Dynamics (NAMD) was used to perform simulations with the Chemistry at Harvard Macromolecular Mechanics 36 (CHARMM36m) force fields (Huang et al., 2017), the TIP3 water model (Jorgensen et al., 1983), and the Beglov and Roux ion parameters (Beglov and Roux, 1994). The switchdist, cutoff, and pairlistdist were set to 9, 10, and 11 Å, respectively. The energy of each model was first minimized by 5,000 steps, followed by three 40-ns production runs at 310 K under the periodic boundary condition and Langevin piston. For every 20 ps, residues 214–225 of LEV-11O (E7a) and residues 186–197 of LEV-11T (E7b) and LEV-11U (E7c) of the simulation products were compared with the corresponding portions of the initial energetically minimized structures along alpha carbons. Then, the average root mean squared deviations (RMSD) of E218 of E7a and E190 of E7b and E7c were calculated. The data were tested by one-way analysis of variance with the Holm-Sidak method for multiple pairwise comparison using SigmaPlot 14.5 (Systat Software). The APBS-PDB2PQR software was used to construct the electrostatic surfaces of E7a, E7b, and E7c at pH 7 and with CHARMM27 force fields using the automatically configured finite difference Poisson-Boltzmann calculations (Jurrus et al., 2018). The electrostatic surface potentials were visualized with red-white-blue coloring, ranging from −10 to 10 kT/e, with an offset of 0.1 and a midpoint of 0.5 by VMD.

### F-actin co-sedimentation assays

Actin was purified from rabbit muscle acetone powder as described (Pardee and Spudich, 1982). Recombinant LEV-11T and LEV-11U were expressed in *E. coli* with extra Ala-Ser after the initiator Met to mimic N-terminal acetylation (Monteiro et al., 1994) using a pET-3d vector with no other tag sequence and purified in the same procedure as described for other LEV-11 isoforms (Barnes et al., 2018). F-actin co-sedimentation assays were performed essentially as described (Ono and Ono, 2020) in F-buffer (0.1 M KCl, 2 mM MgCl_2_, 20 mM HEPES-KOH, pH 7.5, 1 mM dithiothreitol), except that the actin-bound portions of LEV-11 isoforms were calculated from depletion of a constant concentration (2 μM) of the LEV-11 isoforms from the supernatants by sedimentation of varying concentrations of F-actin. Ultracentrifugation was performed at 42,000 rpm (200,000 × g) for 20 min using a Beckman 42.2Ti rotor. Only the top halves of the supernatants were taken and analyzed as supernatant fractions. The rest of the supernatants were carefully removed, and remaining pellets were analyzed as pellet fractions. Proteins were analyzed by SDS–PAGE (12% acrylamide gel) with molecular weight markers (Nacalai United States, catalog no. 29458–24) and staining with Coomassie Brilliant Blue R-250 (National Diagnostics). Percentages of bound LEV-11T or LEV-11U (*P*_*bound*_) were determined densitometrically as (1-*D*_*x*_ */D*_*0*_)x100, where *D*_*x*_ is band density in the supernatants at *x* μM actin and *D*_*0*_ is band density in the supernatants in the absence of actin. Dissociation constants (*K*_*d*_) were calculated using SigmaPlot 14.5 by fitting the data to an equation: *P*_*bound*_ = (*P*_*bound-Max*_ x [actin])/(*K*_*d*_ + [actin]), where *P*_*bound-Max*_ is a maximum *P*_*bound*_ value and [actin] is an actin concentration.

### Transgenic expression of GFP-LEV-11T and GFP-LEV-11U

The cDNA for LEV-11T or LEV-11U was cloned in-frame at the 3’ end of the GFP sequence of pPD-118.20 (provided by Dr. Andrew Fire, Stanford University) containing the *myo-3* promoter that is active in the body wall muscle (Okkema et al., 1993), and injected into the syncytial gonad to generate transgenic animals with extrachromosomal arrays as described (Mohri et al., 2006). The strains generated and used in this study are ON378 *ktEx257 [myo-3p::GFP::LEV-11T]* and ON375 *ktEx254 [myo-3p::GFP::LEV-11U]*. Live worms were anesthetized in M9 buffer containing 0.1% tricaine and 0.01% tetramisole for 30 min, mounted on 2% agarose pads for observation. Staining of worms with ATTO594-phalloidin (Millipore Sigma) was performed as described (Ono, 2001; 2022). Samples were observed by epifluorescence using a Nikon Eclipse TE2000 inverted microscope (Nikon Instruments, Tokyo, Japan) with a CFI Plan Fluor ELWD 40x (NA 0.60) objective. Images were captured by a Hamamatsu ORCA Flash 4.0 LT sCMOS camera (Hamamatsu Photonics, Shizuoka, Japan) and processed by NIS-Elements (Nikon Instruments) and Adobe Photoshop CS3.

### Generation and analysis of an E7c mutant, lev-11(syb4266)

Insertion of a STOP-IN cassette of 43 bp (Wang et al., 2018) to *lev-11* E7c by CRISPR/Cas9-mediated genome editing was performed by SunyBiotech Co., Ltd (Fu Jian Province, China) to generate PHX4266 *lev-11(syb4266)*, which was outcrossed with N2 wild-type three times to generate ON387 *lev-11(syb4266)*. Genotyping was done by single-worm PCR using forward and reverse primers (GAA​GAG​GAG​TTG​CGC​GTC​GTT​GG and ACC​TCC​TTC​TGG​AGC​TTC​TGG​AC) that amplify a genomic fragment [851 bp for wild-type or 894 bp for *lev-11(syb4266)*] containing E7c, followed by digestion with *Nhe* I and agarose electrophoresis. Staining of worms with ATTO594-phalloidin (Millipore Sigma) was performed as described (Ono, 2001; 2022). Worm motility was determined by counting swinging motions of worms in M9 buffer as described (Epstein and Thomson, 1974). Brood size was determined by isolating single L4 worms in individual plates and counting number of progeny until no live worms were produced. The data were tested by Student’s t-test using SigmaPlot.

## Data Availability

The datasets presented in this study can be found in online repositories. The names of the repository/repositories and accession number(s) can be found below: https://www.ncbi.nlm.nih.gov/genbank/, OQ473578.

## Abbreviations

E7, exon 7; F-actin, filamentous actin; GFP, green fluorescent protein; MD, molecular dynamics; RMSD, root-mean-square deviations; Tpm, tropomyosin.
